# Nephrotic syndrome associated with solid malignancies: a systematic review

**DOI:** 10.1186/s12882-024-03632-9

**Published:** 2024-07-04

**Authors:** Shuo Liu, Yuchen Wan, Ziyu Hu, Zhixue Wang, Fenye Liu

**Affiliations:** 1 Department of Traditional Chinese Medicine, Shandong Provincial Hospital Affiliated to Shandong First Medical University, No. 324 Jingwuweiqi Road, Jinan, 250021 China; 2 The First Faculty of Clinical Medicine, Shandong University of Traditional Chinese Medicine, Jinan, China; 3 Department of Traditional Chinese Medicine Internal Medicine, Lianyungang Maternal and Child Health Hospital, Lianyungang, China; 4 Department of Gynaecology, People’s Hospital of Dongying, No. 317 South Dongcheng 1st Road, Dongying, 257091 China

**Keywords:** Nephrotic syndrome, Paraneoplastic nephrotic syndrome, Paraneoplastic syndrome, Solid malignancies, Malignancy

## Abstract

**Background:**

Nephrotic syndrome (NS) can occur as a paraneoplastic disorder in association with various types of carcinoma. However, paraneoplastic nephrotic syndrome (PNS) is often misdiagnosed as idiopathic nephrotic syndrome or as an adverse effect of oncology treatment, leading to delayed diagnosis and suboptimal treatment. The characteristics of NS associated with solid malignancies are not yet elucidated. We systematically summarized the clinical data for 128 cases of NS combined with solid malignancies with the aim of informing the clinical management of PNS.

**Methods:**

We searched the PubMed database for articles published from the date of inception through to October 2023 using the following keywords: “cancer” or “malignant neoplasms” or “neoplasia” or “tumors” and “nephrotic syndrome”, “nephrotic” or “syndrome, nephrotic”. All data were extracted from case reports and case series, and the extraction included a method for identifying individual-level patient data.

**Results:**

A literature search yielded 105 cases of PNS and 23 of NS induced by cancer therapy. The median age at diagnosis was 60 years, with a male to female ratio of 1.8:1. In patients with PNS, manifestations of NS occurred before, concomitantly with, or after diagnosis of the tumor (in 36%, 30%, and 34% of cases, respectively). Membranous nephropathy (49%) was the most prevalent renal pathology and found particularly in patients with lung, colorectal, or breast carcinoma. Regardless of whether treatment was for cancer alone or in combination with NS, the likelihood of remission was high.

**Conclusion:**

The pathological type of NS may be associated with specific malignancies in patients with PNS. Prompt identification of PNS coupled with suitable therapeutic intervention has a significant impact on the outcome for patients.

## Introduction

Cancer is the second leading cause of death worldwide, surpassed only by cardiovascular disease, and poses a substantial threat to public health and societal development [1]. Paraneoplastic syndromes, which manifest clinically in approximately 7–10% of cancer patients [2], often precede detection of an occult malignancy, and their consequences may be more serious than those of the primary tumor [3]. Nephrotic syndrome (NS) is a rare but recognized paraneoplastic disorder. Patients with this syndrome have a group of clinical manifestations that include massive proteinuria, hypoproteinemia, hyperlipidemia, and edema [4], which could indicate a paraneoplastic process or be a result of chemotherapy [5]. Paraneoplastic nephrotic syndrome (PNS) often presents a diagnostic challenge for physicians because it can be difficult to differentiate from idiopathic nephrotic syndrome, which is encountered more frequently, and the adverse effects of treatment for cancer. A prompt and accurate diagnosis is crucial for effective management of NS and any associated malignancy.

Numerous studies have established a definitive temporal association between the onset of NS and clinical emergence of tumors in patients with PNS [6–8]. Typically, remission of the underlying malignancy correlates with a marked decrease in proteinuria, while a resurgence or metastasis of the cancer often precipitates an increase in proteinuria. Tumor antigens and/or antitumor antibodies are frequently detected in the podocyte/subepithelial interstitial spaces during a renal biopsy [9]. Among the hematological malignancies, Hodgkin’s lymphoma is the one most frequently associated with NS, generally by minimal change nephropathy (MCN) [10]. The pathogenesis of NS in Hodgkin’s lymphoma appears to stem from the deleterious effects of tumor lymphocyte byproducts on the glomerulus as opposed to accumulation of immune complexes within the glomerulus [11]. However, solid malignancy-associated NS has not been systematically reviewed, and the pathophysiology remains unclear.

In this study, we systematically reviewed the clinical features, histopathology, relevant laboratory tests, treatment approaches, and prognosis for 128 published cases of NS associated with solid malignancy with the aim of informing the clinical management of PNS.

## Materials and methods

### Data sources and patients

We searched the PubMed database for articles published from the date of inception through to October 2023 using the following search terms: “cancer” or “malignant neoplasms” or “neoplasia” or “tumors” and “nephrotic syndrome”, “nephrotic” or “syndrome, nephrotic”. No language or publication status restrictions were imposed.

**Fig. 1:**
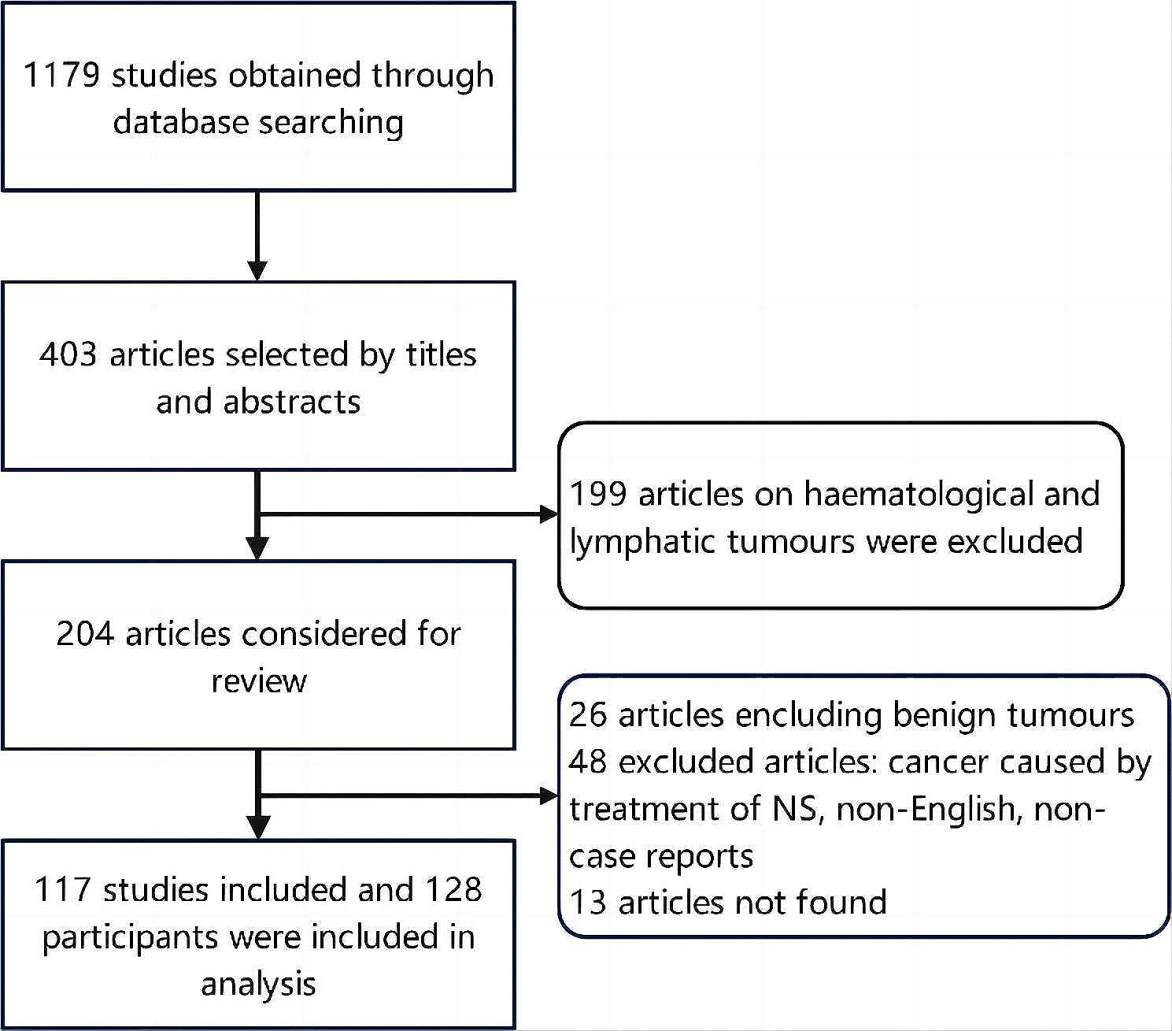
Flow chart showing the literature selection process

All enrolled cases or case series were confirmed based on the following criteria: diagnosis of both NS and solid malignancy and a detailed description of the diagnostic process provided. A flow chart showing the identification of cases and the reasons for exclusions is provided in Fig. 1.

### Study selection, data extraction

Reviews were performed by two independent reviewers (SL and YC). Discrepancies between the two reviewers were solved by asking a third author to review the problematic articles and reach consensus.

Data was collected on the following demographic, clinical, and laboratory variables: country; sex; age at the time of diagnosis; type of solid malignancy; the order of and interval between onset of the two diseases; laboratory values (24-hour urine protein, serum albumin, and serum creatinine); complications of NS (mainly thrombosis, hypertension, and acute renal failure); pathology result of renal biopsy; types of treatment for NS and cancer; interval between initiation of antineoplastic therapy and onset of NS; and clinical outcomes.

### Quality and risk of bias assessment

The quality of the included case reports and series was assessed independently by two authors (SL and YC) using the Joanna Briggs Institute’s(JBI) Critical Appraisal Checklist for Case Reports. The quality of the included studies was variable, and in a small number, it was not possible to extract sufficient data pertaining to the main outcome for inclusion. Additionally, there was variation with interventions used, reporting of outcome measures and timing of outcome measurement. Furthermore, sensitivity analyses were conducted to verify the key findings. This analysis aims to ascertain the stability and reliability of our conclusions by excluding lower-quality case reports and retaining those of higher quality to observe if and how the results change.

### Statistical analysis

Continuous variables were tested for normality of distribution using a onesample Kolmogorov–Smirnov test. Continuous variables with a normal distribution are presented as the mean ± standard deviation and those with a nonnormal distribution are reported as the median (interquartile range [IQR]). Differences between the groups were examined for statistical significance using the chi-squared test. All statistical analyses were performed using Statistical Package for the Social Sciences software (version 25.0; IBM Corp., Armonk, NY, USA). A *P*-value ≤ 0.05 was considered to be statistically significant.

## Results

**Fig. 2:**
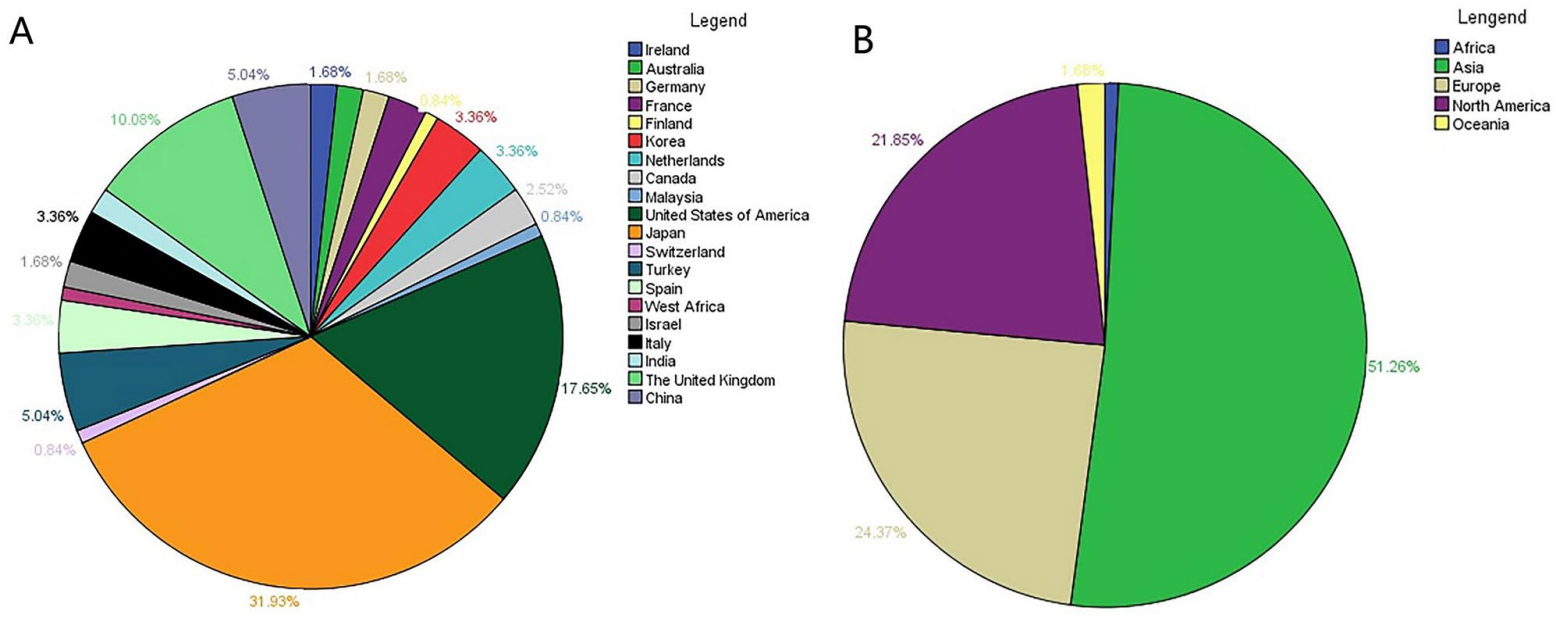
Distribution of cases according to country and continent. (**A**) Percent distribution by country (%). (**B**) Percent distribution by continent

The literature search identified 117 articles that included 128 eligible patients with NS associated with solid malignancy. One hundred and five patients were diagnosed to have PNS and the remaining 23 developed NS as a consequence of cancer therapy. The median patient age was 60 years (range 7–84; IQR 5, 68). Information on sex based on 127 patients showed a male to female ratio of 1.8:1. One hundred and nineteen patients with specific information on region available were from 20 countries and 5 continents. The top three countries were Japan (*n* = 38, 31.93%), the US (*n* = 21, 17.65%), and the UK (*n* = 12, 10.08%). Asia was the continent with the majority of cases (51.26%), with only one case reported from Africa (Fig. 2).

### Clinical features

#### Paraneoplastic nephrotic syndrome

Patients with PNS were separated into three groups based on the interval between onset of the two diseases (NS occurring before, after, or simultaneously with solid malignancy) (Table 1). NS presented as the initial symptom before detection of a solid malignancy in 38 patients (36.2%), with 22 of these cases providing a specific onset time. The median interval between the two diseases was 9.5 months (IQR 5.8, 14.0). NS was identified subsequent to the diagnosis of cancer in 36 patients (34.3%). The interval between diagnoses was reported for 12 of these patients (median 9 months, IQR 6, 36). In the group of patients in whom a solid malignancy was detected first, development of NS was combined with recurrence and metastasis of the primary cancer in 7 patients; the remaining 31 patients (29.5%) received a concurrent diagnosis of NS and solid malignancy.

Quantifiable urine protein levels were documented for 87 of the 105 cases of PNS; the median value was 7.90 g/day (IQR 4.73, 14.45). Sixteen of 105 patients had proteinuria levels represented by “+,” with 13 patients showing 4 + proteinuria and 3 patients showing 3 + proteinuria. Exact data were not provided for the remaining two patients, but the data indicated that proteinuria exceeded 3.5 g/day. Serum albumin levels in patients with PNS were normally distributed. Eighty-nine of the 105 cases had serum albumin levels recorded; the mean level was 1.99 ± 0.61 g/dl. Only 59 of the 105 patients had creatinine levels recorded; the median serum creatinine level was 1.11 mg/dl (IQR 0.86, 2.30). When comparing the three groups, the cohort in which NS preceded the diagnosis of solid malignancies had lower urine protein levels and higher serum albumin levels. Among the cases with detailed data on complications, the group in which NS occurred before the solid malignancy had the highest incidences of hypertension (10/38) and thrombosis (3/38).

**Table 1:** Clinical characteristics of patients with PNS

	NS before solid malignancies (*n* = 38, 36.2%)	NS after solid malignancies (*n* = 36, 34.3%)	NS and solid malignancies simultaneously (*n* = 31, 29.5%)	Total (*n* = 105)
Gender	Men:21 Women:17	Men:24 Women:12	Men:23 Women:7 Unknown:1	Men:68 Women:36 Unknown:1
Age at onset	59 IQR 52 to 64	57 IQR 50 to 68	65 IQR 50 to73	60 IQR 52 to 68
Interval time (months)	9.5 IQR 5.8 to 14	9 IQR 6 to 36		
Urinary protein (g/day)	7.35 IQR 4.45 to 13.40	9.45 IQR 4.22 to 16.25	8.19 IQR 5.90 to 14.68	7.90 IQR 4.73 to 14.45
Serum albumin (g/dl)	2.09 ± 0.58	1.93 ± 0.64	1.92 ± 0.62	1.99 ± 0.61
Creatinine (mg/dl)	1.25 IQR 0.80 to 2.30	0.90 IQR 0.80 to 1.40	1.80 IQR 0.90 to 3.70	1.11 IQR 0.86 to 2.30
Hypertension	10/38	3/36	7/31	20/105
Thrombotic event	3/38	1/36	1/31	5/105

Abbreviations: IQR: interquartile range; NS: nephrotic syndrome

#### NS induced by cancer therapy

Patients with NS induced by cancer therapy were categorized into four groups based on their cancer treatment regimen (Table 2). Information on the interval between the start of cancer therapy and onset of NS was available for 16 of 23 cases and showed significant temporal disparities across the treatment groups. The median interval for 7 of the 14 patients who received targeted therapy (*n* = 14) was 240 days (IQR 70, 600). NS developed in six patients who were receiving immunotherapy within a median interval of 105 days (range 18–540; IQR 47, 270). Two of the patients receiving chemotherapy developed NS at 84 days and 240 days, giving an average interval of 162 days. Furthermore, one case of NS emerged at 1825 days following treatment for bone metastases.

The urine protein level was quantifiable for 12 of the 23 patients with NS induced by cancer therapy; the median value was 6.45 g/day (IQR 3.63, 14.08). The urine protein level was available for 6 of the 14 patients who received targeted therapy and had a median value of 6.45 g/day (IQR 3.63, 9.53). Only three of the six patients in the immunotherapy group had definite urinary protein levels available (mean, 10 g/day). The two patients in the chemotherapy subgroup had a mean urinary protein level of 15.75 g/day. A further patient undergoing treatment for bone metastases had a urinary protein level of 4.6 g/day.

Definitive serum albumin levels were available for 21 of the 23 patients with NS induced by cancer therapy and were normally distributed. The mean serum albumin level was 2.11 ± 0.63 g/dl overall, 2.33 ± 0.62 g/dl in the targeted therapy group, 1.9 ± 0.56 g/dl in the immunotherapy group, and 2.1 g/dl in the chemotherapy group. One patient who was receiving treatment for bone metastases had a notably low serum albumin level of 0.9 g/dl.

Creatinine levels were recorded for 17 of 23 patients with NS induced by cancer therapy; the median value was 1.14 mg/dl (IQR 1.00, 1.79). Among these 17 patients, 6 received immunotherapy, 14 received targeted therapy, 1 received treatment for bone metastases, and 2 were treated with chemotherapy.

Hypertension was observed in nine of the 14 patients in the targeted therapy group, all of whom received an anti-vascular endothelial growth factor agent. Acute kidney injury manifested in three individuals in the immunotherapy group, all of whom were treated with immune checkpoint inhibitors (ICIs).

**Table 2:** Clinical characteristics of patients with NS induced by cancer therapy

	Targeted therapy (*n* = 14)	Immunotherapy (*n* = 6)	Chemotherapy (*n* = 2)	Bone metastasis treatment (*n* = 1)	Total (*n* = 23)
Gender	Men:6 Women:8	Men:6 Women:0	Unknown:1 Women:1	Women:1	Men:12 Women:10 Unknown:1
Age	64 ± 13.8	61 ± 10.0	53	57	62 ± 12.3
Interval days^a^	240 IQR 70 to 600	105 IQR 47 to 270	162	1825	165 IQR 62 to 532
Urinary protein (g/day)	6.45 IQR 3.63 to 9.53	10	15.75	4.6	6.45 IQR 3.63 to 14.08
Serum albumin (g/dl)	2.33 ± 0.62	1.9 ± 0.56	2.1	0.9	2.11 ± 0.63
Creatinine (mg/dl)	1.1 IQR 1.00 to 1.69	2.24 IQR 1.42 to 2.87	1.1	1.97	1.14 IQR 1.00 to 1.97
Hypertension	9/14	2/6		1/1	12/23
Acute kidney injury		3/6	1/2		4/23

^a^Interval between initiation of anticancer therapy and onset of nephrotic syndrome. Abbreviation: IQR: interquartile range

### Paraneoplastic nephrotic syndrome: relationship between renal pathology and type of cancer

Eighty-two of the patients identified in the literature received a definitive histopathological diagnosis. We cataloged the type of renal pathology occurring with a frequency of greater than four and calculated the prevalence of cancers within these renal pathologies (Table 3). Membranous nephropathy (MN) was the predominant type of NS pathology (48.8%), followed by MCN (21.9%), membranoproliferative glomerulonephritis (12.1%), and focal segmental glomerulosclerosis (FSGS, 9.8%). Other rare pathological types included amyloidosis (5.0%) and fibrillary glomerulonephritis (2.4%). Detailed information on the quantitative distribution according to type of primary cancer are shown in Table 4. The primary tumors were most frequently gastrointestinal (26.3%), followed by respiratory (21.1%), genitourinary (18.4%), gynecological (13.1%), and thymic (10.5%).

In the context of MN, lung cancer (7/40), colorectal cancer (6/40), and breast cancer (5/40) emerged as the three most prevalent solid malignancies. In contrast, thymic carcinoma (5/16) had a higher frequency in patients with MCN. Four patients in the membranoproliferative glomerulonephritis group were diagnosed to have renal cell carcinoma (*n* = 2) or lung cancer (*n* = 2). Two patients in the FSGS group developed thymoma and two developed lung cancer.

The remaining four cases of renal amyloidosis occurred in patients with synchronous carcinoma of the stomach and bladder, medullary thyroid carcinoma, gastrointestinal mesenchymal tumor, or lung cancer. Two cases of fibrillary glomerulonephritis were reported (one in a patient with gastric carcinoma and the other in a patient with hepatocellular carcinoma).

**Table 3:** Distribution of types of solid malignancy according to pathological type of nephrotic syndrome

	Urological tumors	Respiratory tumors	Digestive tumors	Gynecological tumors	Others
MN (*n* = 40)	5(12.5%)	10^a^(25.0%)	13^b^(32.5%)	7^c^(17.5%)	5(12.5%)
MCN (*n* = 18)	4(22.2%)	2(11.1%)	4(22.2%)	1(5.6%)	7^d^(38.9%)
MPGN (*n* = 10)	4^e^(40%)	2^f^(20%)	3(30%)	0	1(10%)
FSGS (*n* = 8)	1(12.5%)	2^g^(25.0%)	0	2(25.0%)	3^h^(37.5%)
Total (*n* = 76)	14(18.4%)	16(21.1%)	20(26.3%)	10(13.1%)	16(21.1%)

^a^Lung cancer (7/10). ^b^Colorectal cancer (6/13). ^c^Breast cancer (5/7). ^d^Thymoma (5/7). ^e^Renal cell carcinoma (2/4), ^f, g^Lung cancer (2/2). ^h^Thymoma (2/3)

Abbreviations: MN: membranous nephropathy; MCN: minimal change nephropathy; MPGN: membranoproliferative

**Table 4:** Types of solid malignancy in patients with paraneoplastic nephrotic syndrome

Types of solid malignancies	Number of cases *n* = 76
Urological tumors	*n* = 14(18.4%)
Renal cell carcinoma	7
Prostate cancer	3
Urothelial carcinoma	3
Embryonal rhabdomyosarcoma of the bladder	1
Respiratory tumors	*n* = 16(21.1%)
Lung cancer	13
Bronchial carcinoid tumor	2
Pulmonary lymphoepithelioma-like carcinoma	1
Digestive tumors	*n* = 20(26.3%)
Colorectal cancer	9
Gastric cancer	4
Esophageal cancer	4
Liver cancer	1
Gastrointestinal stromal tumor	1
Undifferentiated carcinoma	1
Gynecological tumors	*n* = 10(13.1%)
Breast cancer	6
Cervical cancer	1
Ovarian cancer	2
Ovarian teratoma	1
Others	*n* = 16(21.1%)
Thymic carcinoma	8
Thyroid carcinoma	2
Pleural mesothelioma	2
Metastatic adenocarcinoma of the appendix	1
Extensive small cell carcinoma of unknown primary etiology	1
Oral cavity cancer	1
Epithelioid hemangioendothelioma	1

### NS induced by cancer therapy: relationship between treatment and pathological type of NS

Renal biopsy was performed in 13 of the 23 patients. The biopsy results are shown in Table 5. The most common type of renal pathology observed in patients receiving anti-vascular endothelial growth factor therapy was thrombotic microangiopathy (3/4), and the biopsy for the remaining patient showed FSGS (1/4). In patients receiving ICIs, the predominant type of kidney pathology was MCN (3/5), followed by one case each of FSGS (1/5) and MN (1/5). The biopsy for a patient who received chemotherapy showed acute tubular injury with duplicated glomerular basement membrane and that for a patient who developed bone metastases showed FSGS.

**Table 5:** Renal pathology in patients with nephrotic syndrome induced by cancer therapy

Cancer therapy	Number of cases (*n* = 13)	Pathologic findings
Targeted therapy	*n* = 5	
BRAF inhibitors	1	Podocyte and endothelial injury^a^
Anti-VEGF therapy	4	TMA^b^/FSGS^c^
Immunotherapy	*n* = 6	
BCG	1	MN
Immune checkpoint inhibitors	5	MCN^e^/FSGS^f^ /MN^g^
Chemotherapy	*n* = 1	Acute tubular injury with duplicated GBM^h^
Bone metastasis treatment	*n* = 1	FSGS^i^

^a^One case received dabrafenib. ^b^Two cases received ramucirumab and one received bevacizumab. ^c^One case received sunitinib. ^e^Two cases received pembrolizumab and one received ipilimumab. ^f^One case received nivolumab. ^g^One case received nivolumab ^h^One case received pegylated liposomal doxorubicin. ^i^One case received pamidronate. Abbreviations: BCG: Bacillus Calmette-Guerin; FSGS: focal segmental glomerular sclerosis; MCN: minimal change nephropathy; MN: membranous nephropathy; TMA: thrombotic microangiopathy; VEGF: vascular endothelial growth factor

### Treatment and outcomes

#### Paraneoplastic nephrotic syndrome

Treatment and outcomes were reported for 101 of 105 patients with PNS. Two of the remaining four patients refused treatment and eventually died, and no treatment or outcomes data were reported for the other two patients. In patients with PNS, antitumor therapy serves as the etiological treatment. Of 101 patients, 92 received antitumor therapy. Treatment of solid malignancy included surgery, radiation treatment, chemotherapy, targeted therapy, and immunotherapy. Symptomatic treatment approaches such as dietary support, diuretics, and intravenous albumin were commonly employed for NS (administered to 39 of 101 patients). Corticosteroid therapy was also commonly administered for renal management (used in 35 of 101 patients). Immunosuppressive agents (i.e., mycophenolate mofetil and cyclosporine) and cytotoxic agents (e.g., cyclophosphamide) were usually selected when corticosteroid resistance or toxicity became problematic. The most common treatment for NS consisted of symptomatic measures (39/101), including dietary support, diuretics, and intravenous albumin. Immunosuppressive agents and dialysis were also reported. Corticosteroids (35/101) were widely used for renal management. Cyclophosphamide, mycophenolate mofetil, and cyclosporine were usually selected when corticosteroid resistance or toxicity was a problem. When the above treatments were ineffective or the patient developed renal failure, dialysis was considered as the primary treatment for NS. Angiotensin-converting enzyme inhibitors and angiotensin II receptor blockers were used more frequently in patients with NS complicated by hypertension. Dipyridamole, aspirin, and HMG-CoA reductase inhibitors were used in patients with concomitant thrombotic events.

Overall response and progression/death rates for the 101 patients with PNS are shown in Table 6. The remission rate was 70.3% in patients treated for cancer only, 81.8% in those treated for both NS and cancer, and 22.2% in those treated for NS only. There was a statistically significant difference in the PNS remission rate among the three treatments (*P* < 0.05). The disease remission rate in patients treated simultaneously for cancer and NS was higher than in those who received the other treatments (*P* < 0.05).

Mortality was 29.7% in the group treated for cancer only. Five of the 11 deaths occurred after remission of NS. The cause of death was progression of cancer or development of cancer-related complications. Mortality was 18.2% in the group treated for both diseases and 77.8% in the group treated for NS only. Mortality was significantly higher in the group treated for NS only than in the other two treatment groups (*P* < 0.05). There was no statistically significant difference in mortality between the group that received treatment for both diseases simultaneously and the group treated for cancer only (*P* > 0.05).

**Table 6:** Response and progression/death rates in patients with paraneoplastic nephrotic syndrome

	Total	Remission	Progression/Death
Only treatment for cancer	37	26(70.3%)	11(29.7%)
Treatment for both diseases simultaneously	55	45(81.8%)	10(18.2%)
Only treatment for NS	9	2(22.2%)^ab^	7(77.8%)^cd^

^a^Statistically significant difference vs. treatment for cancer only. ^b^Statistically significant difference vs. treatment for both diseases simultaneously. ^c^Statistically significant difference vs. treatment for cancer only. ^d^Statistically significant difference vs. treatment for both diseases simultaneously

#### NS induced by cancer therapy

The final outcome was reported for 19 of the 23 patients with NS induced by treatment for cancer. Five of these patients died. The implicated drug was discontinued in all patients because of NS and/or other adverse effects. Except for one patient with cetuximab-associated NS, cessation of the presumed culprit drug led to significant improvement in the NS. In the immunotherapy group, two of the five patients with NS induced by ICIs were re-challenged with ICI therapy after a period of cessation. One patient who had previously been treated with pembrolizumab monotherapy was re-challenged with a combination of ipilimumab and nivolumab immunotherapy and achieved profound remission of the primary cancer without emergence of new toxicities. Another patient treated with ipilimumab experienced a recurrence of NS after remission and subsequently died as a result of progression of the underlying cancer.

### Impact of sensitivity analysis on review findings

The demographic characteristics, diagnostic processes, and treatments in the included case reports are reported comprehensively. However, there were deficiencies in reporting relevant laboratory tests and the time intervals from disease onset, with some data missing. To assess the robustness of our findings, we conducted a sensitivity analysis by excluding case reports deemed to be of lower quality. Quality assessment was based on predefined criteria including the clarity of diagnostic criteria, completeness of patient information and laboratory tests, and the detail provided on treatment outcomes. The initial analysis included 97 case reports of PNS, with the sensitivity analysis retaining 44 reports after excluding 53 lower-quality articles. Table 7 illustrated the comparative statistics of the key outcomes before and after applying the sensitivity analysis, highlighting the changes observed in each parameter. It showed an increase in 24-hour urinary protein values when lower-quality reports were excluded, suggesting more consistent reporting of severe cases in higher-quality reports. The estimated improvement rate after treatment for NS only decreased from 22% in the initial dataset to 0% in the refined dataset, underscoring the potential overestimation of treatment efficacy for NS only in lower-quality reports.

**Table 7:** Results of sensitivity analyses

Outcome Metric	Initial Analysis (All Reports)	Refined Analysis (High-Quality Reports)	Change (%)
Urinary protein (g/day)	7.90	9.43	+ 19%
Average Serum Albumin (g/dL)	1.99	1.97	+ 1%
Creatinine (mg/dl)	1.11	1.16	+ 5%
Improvement Rate for PNS(%)			
Only treatment for cancer	70.3%	70.5%	+ 0.5%
Treatment for both diseases simultaneously	81.8%	76%	-5.8%
Only treatment for NS	22.2%	0	-22%

## Discussion

Kidney damage is common in cancer patients. It has been reported that over 50% of patients with cancer develop mild or severe kidney damage [12]. Moreover, the risk of malignancy in patients with kidney disease is significantly higher than that in the general population. The 5-year risk of any type of cancer has been reported to be 4.7% in patients with NS, an increase of 73% when compared with that in the general population [13]. These data highlight a bidirectional relationship between tumor development and kidney disease. Various tumor-related mechanisms cause kidney damage. Direct causes include destruction of kidney structure by a renal tumor, invasion of the kidney by an extrarenal tumor, and compression of the renal artery and ureter. Indirect causes include tumor lysis syndrome, nephropathy caused by light chain casting in multiple myeloma, and kidney damage induced by antitumor therapies, as well as the abnormal products of tumor cells, including tumor antigens and other unidentified tumor products [14]. The abnormal tumor cell products present as a paraneoplastic symptom that is not directly associated with tumor burden, invasion, or metastasis. To the best of our knowledge, paraneoplastic glomerular disease usually presents as NS [15]. In this study, we focused on the clinical features of PNS, with the intention of providing a reference for clinical practice.

Our study included 128 patients with solid malignancies and NS, of whom 105 had PNS. PNS may appear after, before, or concurrently with diagnosis of malignancy and is more likely to be considered when it occurs after or concurrently with the cancer diagnosis. However, NS can present as the initial clinical manifestation of an underlying malignancy. In this study, 38 patients were identified to have NS before diagnosis of cancer. In the absence of initial evidence of a solid malignancy, this population is prone to being misdiagnosed as having idiopathic nephrotic syndrome. Failure to correctly identify PNS may result in ineffective and potentially harmful treatments [16]. Establishing a temporal link between the two diseases may aid in diagnosing these patients. Some researchers have suggested that screening for tumors should not be neglected in the 6–12 months after onset of NS [17, 18]. Consistent with previous expert opinion [18], our findings indicate that cancer was diagnosed about 9.5 months after diagnosis of NS. The cases summarized in this study were significantly more likely to be male. The types of cancer that develop in patients with kidney disease show a significant difference in sex distribution. Liver, bladder, and kidney cancers are the three most common malignancies in male patients with end-stage renal disease, while bladder, kidney, and breast cancers are more common in female patients [19]. The cases summarized here ranged in age from 7 to 84 years, with a median age of 60 years. Given that the prevalence of cancer in patients over the age of 60 years with NS exceeds 20% [20], older men may be at higher risk of developing PNS. Therefore, it is reasonable to routinely screen for cancer in older men with a recent diagnosis of NS.

Given the occult nature of certain tumors in their early stages, conventional tumor biomarkers were not continuously monitored in the majority of our reported cases. Six cases were monitored for tumor biomarkers and renal function in parallel during treatment [21–26]. In all six cases, the symptoms of NS gradually improved as the relevant serum tumor biomarker levels decreased. Continuous monitoring of conventional tumor biomarkers might be useful for evaluation of the efficacy of treatment and the prognosis of PNS. Although our findings indicate a close relationship between NS and malignancy, conventional tumor biomarkers could not be used alone as diagnostic and screening indicators for PNS. Furthermore, given the limitations of conventional renal biopsies and the fact that tumor antigens are rarely isolated from glomeruli [27], it is essential to find reliable serological biomarkers to differentiate between idiopathic nephrotic syndrome and PNS. Novel specific serum markers have been investigated. Phospholipase A2 receptor (PLA2R) was identified as a significant pathogenic podocyte antigen involved in idiopathic MN and has been suggested to be a tumor suppressor [28, 29]. PLA2R-associated MN accounts for approximately 70–80% of idiopathic cases of MN [30]. Patients with MN in whom serum anti-PLA2R antibodies are detected might not have associated malignancies [31]. In contrast, MN associated with thrombospondin type 1 domain-containing 7 A (THSD7A) has been linked to an increased risk of malignancy and is predominantly expressed in PLA2R-negative cases [32]. THSD7A staining of renal biopsies was strongly correlated with positive THSD7A serum antibodies [33]. Therefore, anti-THSD7A antibody in serum might act as a marker for paraneoplastic MN. Recent findings suggest that neural epidermal growth factor-like 1 protein (NELL-1)-positive MN in renal biopsies is strongly correlated with malignancy and may have a role similar to that of THSD7A [34]. However, further studies are required to elucidate whether the anti-NELL1 antibody titer in serum correlates with underlying malignancy. Patients with MN who are negative for anti-PLA2R antibodies and positive for anti-THSD7A antibodies in serum should be further evaluated for cancer. Further development of serum and glomerular biomarkers with a high degree of sensitivity and specificity is required in terms of screening for other pathological types of NS.

PNS was milder (with higher serum albumin and lower proteinuria levels) in the group in which NS was detected first than in the group in which the tumor was detected first and the group in which the two diseases were detected simultaneously. The severity of NS was positively correlated with exacerbation of the underlying malignancy [35]. When NS is diagnosed in these patients, the cancer may still be in the early stages with a lower tumor burden and fewer associated antigen-antibody complexes. Nevertheless, the prevalence of hypertension and thrombotic events in this group was higher than in the other two groups. Hypertension in patients with NS may result from chronic edema or some underlying cause of nephropathy [36]. Previous investigations have found that hypertension is the most common cardiovascular comorbidity in cancer patients, with a prevalence of up to 37% [37]. Considering the milder symptoms of edema in the group in which NS was detected, we assume that the higher prevalence of hypertension may be secondary to the underlying malignancy, anticancer treatment, or other causes independent of cancer and NS. Approximately 25% of individuals with cancer-related MN experience thrombotic events [38], and previous cancer and hypertension are independent risk factors for thromboembolism in patients with NS. Therefore, we propose that thrombotic events may serve as a clinical indicator warranting suspicion of underlying malignancy in patients with NS.

The pathological type of NS may be related to the type of tumor, and this association may be more pronounced for specific cancers. Renal glomerulopathy in PNS usually manifests as MN in patients with solid malignancy, especially in patients with lung or gastrointestinal tract cancers [39]. In our study, MN accounted for almost half (49%) of all patients receiving renal biopsies. Of the 13 patients with lung cancer-associated NS, seven (54%) were diagnosed with MN. Five (56%) of nine patients with colorectal cancer had combined MN. It is also worth noting that five (83%) of the six cases of breast cancer had a diagnosis of MN. Furthermore, MCN was frequently observed in glomerular lesions among patients with thymoma. Renal biopsies in five (63%) of the eight patients with thymoma showed MCN. When the above-mentioned tumors are diagnosed, our findings may provide direction for the treatment of NS in patients who are not eligible for renal biopsy. No significant correspondence was found between other pathological types of NS and tumor types, mainly because of the limited number of cases reported. Therefore, more extensive studies in larger sample sizes are warranted.

Typically, management of PNS centers primarily on addressing the underlying tumor. In 25% of patients with tumor-related NS, successful treatment of the cancer was followed by remission of NS [40]. In the present study, the improvement rate was higher in patients who received concurrent treatment for both conditions than in those who received treatment solely for a tumor or NS. In addition to symptomatic treatment, corticosteroids were frequently used in the treatment of NS. The selection and duration of hormone therapy, as well as the choice and utilization of cytotoxic agents, should be tailored to the patient’s renal function, age, glomerulopathy-related contraindications, and the specific pathological type. Furthermore, clinicians should focus on prevention of thromboembolic events and hypertension in these patients.

Antineoplastic drugs, including cytotoxic agents, targeted agents, and immunomodulators, had a similar role in inducing and aggravating kidney damage in cancer patients, which was confirmed by the close temporal relationship between the onset of NS and administration of certain agents. In our study, NS was induced in 23 patients after a period of use of antineoplastic drugs; and 22 of these patients showed significant remission of NS after cessation of these agents. Anti-angiogenic targeted therapies and ICIs, which have become important anticancer treatments in recent years, have an uncertain impact on NS. It is a challenge to manage renal adverse reactions caused by antineoplastic agents.

## Conclusion

In conclusion, NS can present as a paraneoplastic syndrome or as an adverse effect of antineoplastic agents in patients with solid malignancies. A definitive diagnosis is required to formulate effective treatment options for NS. The diagnosis of PNS becomes more challenging when NS precedes the onset of malignancy. It is difficult to decide which patients should be screened for malignancies. Our findings indicate that older male patients with NS may have a higher risk of underlying cancer. A negative anti-PLA2R antibody result and a positive anti-THSD7A antibody result in serum may prompt the search for an underlying malignancy. Based on the definitive diagnosis, patients treated for both diseases simultaneously have a higher remission rate. We also found that the pathological type of NS may be associated with specific tumors in patients with PNS. This hypothesis requires further investigation.

## Data Availability

All data generated or analyzed during this study are included in this article. Further enquiries can be directed to the corresponding author.

## Abbreviations

FSGS: Focal segmental glomerular sclerosis
MCN: Minimal change nephropathy
MN: Membranous nephropathy
MPGN: Membranoproliferative
NELL-1: Neural epidermal growth factor-like 1 protein
NS: Nephrotic syndrome
PLA2R: Phospholipase A2 receptor
PNS: Paraneoplastic nephrotic syndrome
THSD7A: Thrombospondin type 1 domain-containing 7 A
